# The International College of Psychosomatic Medicine – a personal history

**DOI:** 10.1186/s13030-016-0076-2

**Published:** 2016-08-24

**Authors:** Jon Streltzer

**Affiliations:** Professor Emeritus of Psychiatry, University of Hawaii School of Medicine, 1356 Lusitana Street-4th Floor, Honolulu, HI 96813 USA

**Keywords:** Psychosomatic medicine, History

## Abstract

This is a history of the International College of Psychosomatic Medicine from 1970 to the present.

## The beginnings

The International College of Psychosomatic Medicine ICPM) was founded in 1970, and the first World Congress on Psychosomatic Medicine was held in Guadalajara, Mexico in 1971. I graduated medical school in 1970. I had not decided on a specialty until my last year, when I discovered psychiatry. I was concerned, however, that most of my medical training would not fully be used in that specialty, and I was attracted to the idea of psychosomatic medicine, treating the whole person. I did residency training at Yale, where the chairman of the department of psychiatry was Morton Reiser, one of the most prominent figures in psychosomatic medicine at that time. He was the editor of the journal, *Psychosomatic Medicine* and would soon become president of ICPM. A key supervisor and mentor during my training was Hoyle Leigh, chief of the consultation–liaison service at Yale-New Haven Hospital that was very advanced for its time. He became an early and important member of ICPM. I was also supervised by Chase Kimball, another future president of ICPM.

**Table 1:** Presidents and Congresses of ICPM

Presidents of ICPM	World Congresses
Eric Wittkower, Canada 1970–1973	1st Guadalajara, Mexico 1971
Mauricio Knobel, Argentina 1973–1975	2nd Amsterdam, Netherlands 1973
Morton Reiser, USA 1975–1977	3rd Rome, Italy 1975
Yujiro Ikemi, Japan 1977–1979	4th Kyoto, Japan 1977
Adam Krakowski, USA 1979–1981	5th Jerusalem, Israel 1979
Jan Bastiaans, Netherlands 1981–1983	6th Montreal, Canada 1981
Chase Kimball, USA 1983–1985	7th Hamburg, Germany 1983
Cairns Aitken, UK 1985–1987	8th Chicago, USA 1985
Juan Lopez-Ibor, Jr., Spain 1987–1989	9th Sidney, Australia 1987
Herbert Weiner, USA 1989–1991	10th Madrid, Spain 1989
Michael von Rad, Germany 1991–1993	11th Brazil –Cancelled
Atara Kaplan deNour, Israel 1993–1995	12th Bern, Switzerland 1993
Hubert Lacey, UK 1995–1997	13th Jerusalem, Israel 1995
Don Byrne, Australia 1997–1999	14th Cairns, Australia 1997
Don Lipsitt, USA 1999–2001	15th Athens, Greece 1999
Tatjana Sivik, Sweden 2001–2003	16th Göteborg, Sweden 2001
George Christodoulou, Greece 2003–2005	17th Waikoloa, Hawaii, USA 2003
Tom Sensky, UK 2005–2007	18th Kobe, Japan 2005
Giovanni Fava, Italy 2007–2009	19th Quebec City, Canada 2007
Jon Streltzer, USA 2009–2011	20th Torino, Italy 2009
Chiharu Kubo, Japan 2011–2013	21st Seoul, Korea 2011
Thomas Wise, USA 2013–2015	22nd Lisbon, Portugal 2013
Antonio Barbosa, Portugal 2015–2017	23rd Glasgow, UK 2015
Fiammetta Cosci, Italy President-elect	Future Congresses – Beijing, China 2017; Florence, Italy 2019

As I understand it, ICPM was formed by a group of international physicians, most of whom were quite prominent in academic circles. Psychosomatic medicine has always been considered a multidisciplinary field, inclusive of physicians of all clinical specialties and other healthcare professionals, such as physical therapists, psychologists, nurses and so forth. I have not been able to determine the extent of nonphysician involvement in the founding of ICPM. I suspect it was relatively minimal.

An anonymous peer reviewer of this manuscript has provided information that the Dutch delegation, including Drs. Jan Bastiaans, Herman Mustaph, Henk Pelser and Professor J.J. Groen, was among those instrumental in the founding of ICPM.

At the time of the founding of ICPM and in the early years, major theoretical summaries and explications of the field were being produced. In a paper entitled “Conceptual Developments in Psychosomatic Medicine: 1939–1969”, Chase Kimball listed ten concepts that described psychosomatic medicine that are as accurate and pertinent today as they were in 1970 [1]. To quote two from that paper: “All illnesses have psychosocial aspects that influence their cause, precipitation, manifestation, course and outcome.;” “The approach to the individual suffering from a specific illness is specific depending on the idiosyncrasy of the patient’s life situation, which includes, in addition to attending to the disease process, attending to the psychological and social correlates.”

The most prolific author of that period summarizing and defining the theory and practice of psychosomatic medicine was founding ICPM member Z. J. Lipowski. His definition, still widely accepted today, was “1) It is the scientific discipline concerned with the study of the relationships of biological, psychological and social determinants of health and disease. 2) It is a set of postulates and guidelines embodying a holistic approach to the practice of medicine. 3) It encompasses consultation–liaison psychiatry.” [2].

During my residency training, I was strongly influenced by the tutelage of my mentor, Hoyle Leigh and the psychosomatic approach to medicine articulated by doctors Kimball and Lipowski. I gained experience in the clinical aspects of psychosomatic medicine as chief resident of the psychiatric consultation–liaison service of Yale–New Haven Hospital, including two consecutive years as liaison to the developing hemodialysis program. At the end of my residency, I joined the American Psychosomatic Society. I then took a position with the new University of Hawaii medical school in Honolulu, Hawaii, and, among other duties, directed the psychiatric consultation service at the Queens Medical Center for over 25 years. Living in a multicultural society, and encouraged by Hoyle Leigh, I soon joined ICPM.

ICPM membership included a subscription to the *Journal of Psychosomatic Research* and a discounted rate for subscription to *General Hospital Psychiatry*. In those days before computers and the Internet, receiving these journals was likely highly valued by the members of ICPM –certainly it was important to my teaching and practice. I had hoped to attend the biennial World Congresses, but due to family and academic responsibilities I was only able to attend the Montréal Congress in 1981. In my recollection, the attendance at that Congress was very large, something like 1000–2000 registrants. It was overall a wonderful experience, not just educational, but also enjoyable because of the academic and social connections formed and the cultural experiences of being in Montréal for the first time. I recall feeling skeptical about some of the psychoanalytically – oriented lectures that were quite different from the approaches to the practice of psychosomatic medicine that I had come to believe in.

## Personal involvement in ICPM

In 1999, the incoming president of ICPM, Don Lipsitt, contacted me about organizing a World Congress in Hawaii. Don did not know me, and I presume he got my name from his former colleague, Byron Eliashof, with whom I was doing a research project on chronic pain in injured workers. I knew of Don as a leader in the field of psychosomatic medicine, a journal editor, teacher and researcher. I had heard him speak at conferences and was very impressed. Flattered by his invitation, I agreed to organize the 2003 Congress, not having any idea what I was getting into. This task would dominate the next four years of my life.

I found out that ICPM had very limited financial resources, no employees to manage things, and could provide nothing toward the development of the Congress, only advice! Furthermore, there was no provision or obligation for ICPM to cover any financial loss that might occur. I did obtain great advice from others who had organized congresses, including Don Lipsitt (USA), Tatjana Sivik (Sweden) and George Christodoulou (Greece). Particularly helpful was Don Byrne from Australia who had organized two world congresses and was then president of ICPM. I set about trying to understand the intricacies of psychosomatic medicine in different parts of the world in order to create a successful World Congress.

I made sure to attend the next ICPM World Congress in 2001 in Gothenburg, Sweden that was organized by Tatjana Sivik. This was a lovely Congress, and I paid close attention to the organization of both the scientific program and the social events.

I knew nothing of destination management companies at that time. Such companies have proliferated in recent years and have developed great expertise in the complexities of organizing international conferences. I needed a manager, and a member of my medical school department administrative staff claimed she had experience organizing conferences. Partly, I chose her because of her reasonable fee, but you get what you pay for. The continuing education department of my hospital in Honolulu, The Queen’s Medical Center, agreed to handle continuing medical education credits and provide some administrative support. It also provided an educational grant of $25,000 that was critical to the start up. Later, I also obtained some funding from pharmaceutical companies, something that has become increasingly difficult for psychosomatic conferences in subsequent years. To take care of the audiovisual needs of the Congress, which became quite complex, I was very fortunate to obtain the services of a specialist in the medical school, Gary Belcher. He was so competent at this, and also as an administrator, that ICPM hired him to be our part-time administrator from 2004 through the present. The location of the Secretariat moved to Honolulu to be with Gary, where it remains to this day. This has provided stability and accountability. Previously, the location of the Secretariat moved with each president.

I formed a local organizing committee of physicians prominent in psychosomatic medicine, cultural psychiatry and administration. I appointed Norman B. Levy, M.D., to be cochair with me of the International Scientific Advisory Committee. Norman was known as the “Father of Psychonephrology ” and had organized many psychonephrology conferences. We appointed 73 people, prominent in many countries and from several disciplines, to this committee.

I deviated from usual practice and selected a five-star beach resort on the Hawaii Island of the state of Hawaii as the Congress venue. This incredible resort hotel offered superb conference facilities at no charge if we filled enough rooms with our attendees. The closest low-price accommodation was 30 min away and there was no public transportation, so, despite the cost, I was hoping the meeting and the venue would be attractive enough to draw sufficient registrants to avoid a penalty charge for the conference rooms. I had great anxiety about this until about 3 weeks before the start of the Congress when enough rooms finally got reserved. The Congress lasted six days, with one day entirely devoted to a circle island tour. The scientific quality of the Congress turned out even better than I had anticipated. We managed to have 14 top-notch plenary speakers, most of whom accepted no honorarium. We had symposia, workshops, free communications and poster sessions. We had a themed dinner in addition to a welcome reception and a gala luau dinner. Despite numerous anxieties and problems along the way, we ended up with almost 600 registrants from 40 countries. Many told me it was the most enjoyable conference they ever attended. At the ICPM business meeting, I was nominated and elected secretary of ICPM. I served two terms as secretary, and then was elected President–elect.

The next major event for ICPM was the 18th World Congress in Kobe, Japan in 2005 organized by Chiharu Kubo and Tomifusa Kuboki. This Congress again attracted attendees from all over the world, and attendance reached a remarkable 1300 registrants due to the great interest in psychosomatic medicine in Asian countries. A highlight for me was meeting Emperor Akihito and Empress Michiko. It was a great honor that the Emperor chose to attend our ICPM Congress. When he arrived just before the opening ceremony, he was escorted up the back stairs by security, and those of us on the ICPM executive board were positioned to greet him. We were told not to shake hands, and instructed how to bow correctly. When he and the Empress came into the foyer where we were standing, he greeted each of us in turn. When he was introduced to me, he said in unaccented English, “You are from Hawaii, aren’t you?” and he shook my hand! Empress Michiko was equally gracious with us. The Emperor’s introductory remarks can be found at http://www.convention-news.co.jp/psycho.htm.

The 19th World Congress took place in 2007 in Québec City, Canada, organized by Louis van Zyl and Fabien Gagnon. Québec city is a delightful, French speaking town, three hours north of Montréal. It is filled with street performers and artists. The Congress was excellent, scientifically and culturally. Unfortunately, attendance was somewhat less than hoped-for.

The 20th World Congress was organized by Giovanni Fava, the editor of the journal, *Psychotherapy and Psychosomatics*. It was held in Torino, Italy in 2009 in the Lingotto Conference Center, a huge complex that had formerly been a factory for manufacturing cars, airplane engines and various appliances. A number of educational courses were added to the syllabus, and there were less than usual social events. Attendance was good, however, but this was the second Congress in a row that failed to come out ahead financially.

The 21st Congress was organized by Kyung Bong Koh and Byung-Il Min in Seoul, Korea in 2011. The venue was unusual and spectacular – the National Museum of Korea. The scientific program was again superb, and the gala dinner featured fantastic food and entertainment by an amazing avant-garde K-Pop group. Attendance was excellent and the Congress was a financial success.

In 2013, the 22nd Congress returned to Europe, organized by Antonio Barbosa in Lisbon, Portugal. Lisbon is a wonderful city. It was easy to get around from the Congress venue, the Lisbon Marriott. There is much to see and do in the areas surrounding Lisbon. Once again the scientific program was of very high quality. Attendance was sufficient so that the Congress broke even.

The 23rd World Congress, held in Glasgow, Scotland, in 2015, was particularly satisfying for me, and a turning point, with several firsts for ICPM. Many cities, convention centers and other venues compete for conferences and group meetings and I had begun to meet with many representatives from various venues several years before. Glasgow had indicated that there was a healthcare professional that might be interested in hosting our Congress there in 2015. Indeed, a bid was submitted and we had competitive bidding for the first time in my memory. Previously, the host organizer was found by word of mouth, usually being an officer of ICPM, and while most congresses were successful, there were mixed results. The submitted bid was more professional than any we had previously seen, and we were very impressed by the support promised by the Glasgow and Scotland visitor industry. The host organizer, Mike Gow, is a dentist, another first for ICPM and an advance for the mission of psychosomatic medicine. The Congress turned out to be very successful scientifically, financially and socially. Attendance by dentists was significant. There were excellent scientific presentations that included dental-related topics such as temporomandibular disorder and dental hypnosis.

Future congresses have been selected, also going through a competitive bidding process, Beijing, China in 2017 and Florence, Italy in 2019. The bidding for the 2019 Congress included four excellent sites submitting detailed proposals!

## Presidents of the college

The presidents of ICPM have each served a 2-year term. They have been innovators in psychosomatic research and have made major contributions to the advancement of the field of psychosomatic medicine.

The first president, Eric Wittkower of Canada, was one of the early pioneers of psychosomatic medicine and a major academic figure at McGill University in Canada. He had done many of the early studies on the influence of emotions on the function of bodily organs in normal and neurotic individuals. Wittkower was succeeded by Mauricio Knobel, a prominent psychoanalyst from Argentina and Brazil. In 1975, Morton Reiser of the United States became president. Succeeding Professor Reiser was Professor Yujiro Ikemi of Japan, a major figure in Japanese psychosomatic medicine. He founded the Department of Psychosomatic Medicine at Kyushu University in Fukuoka.

In 1979, Adam Krakowski of Plattsburgh, New York became the fifth president of ICPM. Dr. Krakowski had been instrumental in helping to organize the College in 1971, along with colleagues from North and South America and Sweden. From 1981 to 1983, Professor Jan Bastiaans of the Netherlands was president of the College. Ever since the Dutch psychiatrist Querido tried to establish a biopsychosocial approach to medicine in the 1950s and 1960s, interest in psychosomatic medicine and consultation-liaison psychiatry has grown considerably in that country. In 1983, Chase Kimball of Chicago, USA became president of the College. Kimball was strongly committed to the development of consultation-liaison psychiatry in the United States and, as an international lecturer on the subject, encouraged those in other countries to pursue the field. Although Kimball was a major participant in and contributor to Congresses from the very first, his beginning illness took its toll on both Kimball and the Congress, resulting in a slump in the College, with a slow recovery.

From 1985 to 1987, Professor Cairns Aitken of Scotland was president of the College He was also editor of the College’s *Journal of Psychosomatic Research*. In 1987, Juan Lopez-Ibor, Jr., of Spain, assumed the presidency. He later became President of the World Psychiatric Association.

The next president of ICPM, from 1989 to 1991, was Professor Herbert Weiner of the United States. Dr. Weiner has been a major figure in the history of psychosomatic medicine, having been president of the American Psychosomatic Medicine and editor-in-chief of the journal *Psychosomatic Medicine*. In 1991, Professor Michael von Rad of Munich, Germany became president of ICPM. His many studies of alexithymia contributed to the clarification as well as the continuing controversy about this interesting topic.

Professor Atara Kaplan deNour of Jerusalem, Israel was the 12th president of ICPM from 1993 to 1995. She had been Chairman of the Department of Psychiatry at Hadassah University Medical School and had a special interest in the psychological aspects of renal disease, a specialized field named by Norman Levy as “psychonephrology.” In 1995, the UK was again represented, this time by Professor Hubert Lacey. It was under Professor Lacey’s presidency that the College’s constitution was completely rewritten to reflect more contemporary requirements. Professor Don Byrne of Australia assumed the presidency from 1997 to 1999. Professor Byrne hosted two memorable Congresses in his country.

Don Lipsitt, USA, was president 1999–2001. Don has had a major impact in the ongoing functioning of ICPM and on the field of psychosomatic medicine in general to this day [3]. Professor Tatjana Sivik became president 2001–2003. She organized the 2001 Congress and helped ICPM regain a stable financial footing. From 2003 to 2005, George Christodoulou from Greece was president. He was also a major figure in the World Psychiatric Association. Tom Sensky, UK, was president 2005–2007. He was a founding Fellow of the Academy of Cognitive Therapy and is an expert on evidence-based practice.

Giovanni Fava, Italy, was president from 2007 to 2009. A noted researcher and editor of the journal *Psychotherapy and Psychosomatics*, he was the prime mover in affiliating that journal with ICPM. From 2009 to 2011, Jon Streltzer was president after serving ICPM for many years as Secretary. Under his guidance, Gary Belcher was hired as administrator and the location of the secretariat moved to Honolulu, Hawaii where it is still located. From 2011 to 2013, Chiharu Kubo of Japan was president. Prof. Kubo was Chair of the Department of Psychosomatic Medicine, Kyushu University and also president of the Asian College of Psychosomatic Medicine for many years. He is currently President of Kyushu University. Professor Kubo was followed by Thomas Wise, USA, 2013–2015. Dr. Wise has long been a scholar and leader in psychosomatic medicine. He was previously president of the American Psychosomatic Society and also the Academy of Psychosomatic Medicine. Antonio Barbosa, Portugal, became president in 2015, after organizing a successful Congress in 2013. In 2017, Fiammetta Cosci, Italy, will become president and will also organize the 2019 Congress (Table 1).

## Conclusion: ICPM – past, present, future – my view

The structure and function of ICPM in the past 20 years has become quite different from what it was in the first 20 years. The original mission of ICPM was to foster and promote psychosomatic medicine throughout the world. The letterhead from a document dated 1972, reveals that the original managing Executive Council consisted of the president, two presidents elect, five vice presidents, one from each continent except Australia, a secretary, an executive secretary–treasurer and six councilors. Currently, ICPM is run by an executive board consisting of the president, the president elect, the immediate past president, a secretary and a treasurer. There is also an advisory board appointed by the president. In recent years, the advisory board has included all past presidents, the organizers of future congresses, and a few other key people in psychosomatic medicine.

In a 1989 document of the agenda of the Executive Council, reports of 13 committees were planned. These committees included audit and finance; child and developmental; constitution/bylaws; education; liaison; long-range planning; membership and recruitment; psychopharmacology; psychosomatic training for physicians; publication; public relations; research; and a nominating committee. This plethora of committees in the early years of ICPM attests to the hopes and goals that ICPM would be a major force in the international development of psychosomatic medicine. The virtual impossibility of members from all over the world meeting between biennial Congresses was too problematic, however, for those committees to function effectively. Currently, ICPM has only three or four committees, and only one, membership, is active. Instead, national and regional societies have become increasingly active in the field of psychosomatic medicine, replacing ICPM in effectively promoting the field.

These changes may actually be a good thing. The field of psychosomatic medicine has grown tremendously and is thriving. It is much too large and complex at this juncture to be governed by an international organization. ICPM has struggled to maintain its relevance. In recent years we have attempted to provide salience in between congresses by having regular newsletters, journal article discussions and educational workshops. These efforts have not proven sustainable, nor have they substantially increased membership.

The biennial world conferences have retained their appeal, however. The world has become much smaller than it was in 1970. Many of us are eager to travel to other countries, develop international contacts and friendships, and share our perspectives. The increasing interest and support from cities and countries to hold international conferences holds promise that we can continue to have successful congresses if we take advantage of such. If ICPM can resist any tendency to become aloof and insular, and increase collaborations with national and regional psychosomatic societies, I believe it will endure and prosper.
